# Impacts of sleep disturbance and work-related life stress on depression among Japanese and Chinese workers

**DOI:** 10.1371/journal.pone.0305936

**Published:** 2024-06-27

**Authors:** Eiko Matsuda, Mariko Kikutani

**Affiliations:** 1 Department of Social Psychology, Faculty of Sociology, Toyo University, Bunkyo-ku, Tokyo, Japan; 2 Institute of Liberal Arts and Science, Kanazawa University, Kanazawa, Ishikawa, Japan; Institute of Mental Health, SINGAPORE

## Abstract

The present study investigated how life stress and sleep disturbance impact depressive symptoms among Chinese (*N* = 185) and Japanese (*N* = 464) workers. Based on a hypothesis that sleep disturbance can cause depression, a statistical model is established, expecting that work-related life stress indirectly increases depressive symptoms by worsening sleep disturbance rather than initiating depression directly. The study also examined the buffering effects of social support on depression. The extent of depressive symptoms, sleep disturbance symptoms (insomnia, hypersomnia, and nightmare), work-related stressors, and available social support were measured. The result revealed that the extent of depression was equivalent for both groups, but the Chinese reported more stress, less social support, and more severe sleep disturbance symptoms than the Japanese. Despite those differences, the statistical model fitted both groups well, suggesting that addressing sleep disturbance at the earliest opportunity can effectively prevent depression onset for workers.

## Introduction

Depression is a major contributor to the global burden of disease, affecting approximately 5% of adults worldwide. Its symptoms are wide-ranging, including anxiety, loss of motivation and interest in life, low self-esteem, and sleep-related problems [1]. A variety of causal factors of this illness have been suggested, such as family history [2], sex [3], and socio-economic status [4, 5]. Exposure to stressful life events [6] is also one of the most investigated risk factors for the illness. In addition, a growing body of research suggests that sleep disturbance, which was traditionally thought to be a symptom of depression, is actually the cause [7, 8].

Among different sleep disorders, insomnia seems to have a particularly strong relationship with depression [see reviews in 9], and many studies have concluded that insomnia symptoms reliably predict the development of depressive disorders [10–15] as well as relapses [16–18]. Among people without depression, individuals with pre-existing sleep disturbance have a significantly higher risk of developing the illness than those without sleep problems [7, 19, 20].

Importantly, it appears that the effect of sleep disturbance on depression is strongly related to the amount of stress experienced by the same person. It is well-established that physiological responses to stress can directly trigger depression [21, 22]. However, stress may affect depression indirectly through sleep disturbance, causing it or worsening symptoms. This interactive relationship between stressful life events and sleep disturbance was implied in a recent longitudinal study by Feldman et al. [23], which investigated predicting factors of psychopathology among emergency medical service providers. They found that poor sleep quality at baseline predicted an increase in depression three months later, while perceived stress levels at baseline did not. This account leads to a hypothesis that the relationship between life stress and depression is mediated by sleep disturbance.

Recently, Matsuda and Kikutani [24] targeted Chinese and Japanese university students and investigated the relationship between depressive symptoms, sleep disturbance (insomnia, hypersomnia, and nightmares), and stressful life events. They tested a hypothetical model predicting that a greater number of life stressors is directly related to increased depressive symptoms, but this relationship was significantly mediated by sleep disturbance, such that the indirect effect of stressors via sleep disturbance was more robust than its direct effect. Their results confirmed the model and suggested that sleep disturbance has a strong relationship with depression. The primary function of stressful events is to increase sleep disturbance rather than increase depressive symptoms directly. Their results also revealed that the prevalence of the three sleep disturbances was slightly different between the two countries, highlighting the importance of cross-cultural investigations on this issue.

The present research followed Matsuda and Kikutani [24] and investigated the depression mechanism among the working population in China and Japan. Although there are considerable variations in working style, this research defines the working population as people whose central activity is working to earn money, contrasting to students whose central activity is studying. Among a variety of potentially stressful events that can trigger depression, work-related stress has been researched more and more in recent years based on the evidence that a substantial part of psychological distress for adults is related to work [25–28]. This line of research includes investigations of an influential theory, the demand-control model, by Karasek and Theorell [29]. According to the theory, a combination of having jobs with high psychological demands and low job control (decision authority and skills discretion) increases the risk of mental illness [26, 30–32].

It is well-established that job-related stress increases the risk of sleep disturbance. Long working hours [33], high psychological demands for work [34], low levels of job control [35, 36], work-related conflict [37], and job insecurity [35] can all contribute to sleep disturbance. Therefore, the relationship between life stress and depression can be mediated by sleep disturbance among the working population.

Following Matsuda and Kikutani [24], the present research was conducted in China and Japan because comparing these two countries should have significant implications for the mental health of people living there, who are native or otherwise. The current research chose these countries because the study samples were easily accessible to the authors. Also, the investigation is urgent due to their extremely active interactions in recent years. According to Japan’s Ministry of Foreign Affairs [38], approximately 745,000 Chinese reside in Japan as of 2021 (25% of all foreign residents). At the same time, China has been the second most popular foreign country Japanese choose to live in after the US. As of 2021, approximately 108,000 Japanese live in China. Most of those immigrants are students and adult workers. Therefore, contrasting depression-related problems in these two countries may inform immigrant workers about their potential mental health issues and how they differ from those in their native culture.

Although China and Japan are close neighbors and have many cultural similarities, such as collectivism [39], their political and socio-economic systems greatly differ, with China being a communist country and Japan being a capitalist one. This difference strongly influences the two countries’ business systems, corporate governance, and working culture [40]. For example, state-owned and state-private hybrid enterprises are common in China [41], while Japan rejects the idea of state ownership [42]. As a result, different Chinese corporations are, in the ultimate sense, united under the state’s power and are working for common goals. To achieve this, Chinese corporations tend to develop rigid hierarchical relations among workers governed by state authorities [43]. Individual workers should regard themselves as a part of the Chinese business system and contribute to the national goals. In contrast, Japanese corporations are much more independent, and harmonious relations among workers within each organization are more valued than efficiently working towards the state’s economic system [44]. These cultural differences are likely to affect how people experience stress at work and consequently impact sleep disturbance and depression symptoms. Matsuda and Kikutani [24] reported that Japanese university students had worse depression and hypersomnia symptoms than Chinese students, while life stress and nightmare symptoms were more prominent for the Chinese. These tendencies appeared to be influenced by the students’ lifestyles in both countries and therefore, the results in this study with workers might be different.

The present research collected data from Chinese and Japanese workers, mostly company employees with full-time and long-term contracts, working in their native country. The participants reported the extent of job stress, sleep disturbance, and depressive symptoms. The data were analyzed using the statistical model proposed by Matsuda and Kikutani [24]. The study considered the level of social support as a newly added variable. The availability of social support has an immense influence on depression. The presence of social support works to lessen the adverse consequences of the illness, and lack of social support can directly increase depressive symptoms [45]. In occupational health, individuals with little social support, in addition to high-strain jobs and low job control, are reported to be most vulnerable to mental illnesses (the demand-control-support model by [46]). The present study examined whether the extent of subjective social support reception influences the interactive relationship between job stress, sleep disturbance, and depression.

Apart from comparing China and Japan, the novel aspects of the present research are 1) considering multiple sleep symptoms and 2) investigating stress, sleep, and depression at once. While the impact of insomnia on depression has been thoroughly investigated [see 9], the relationship between depression and hypersomnia or nightmares is not as commonly analyzed. Also, no study has directly compared the prevalence of these two symptoms among Chinese and Japanese workers within the authors’ knowledge. Regarding 2), it is not very common to investigate the three variables at once, although a large number of studies have attended to either the relationship between depression and stress [21, 22] or between depression and sleep [7–20]. The present research focused on the mediation effect of sleep disturbance on the relationship between depression and work stress, which is a relatively novel attempt.

## Methods

### Participants

Participants were 674 workers from China or Japan working in their native countries at the time of the study. They were recruited to the study either directly by the authors or indirectly through their workmates whom the authors contacted (see the data collection and ethical considerations for the details). They participated voluntarily and received no reward. Twenty-five of them did not properly complete the study, so they were removed from the sample. The reminders were 185 Chinese (74 men, 107 women, 4 unreported, mean age = 33.95, *SD* = 8.73) and 464 Japanese (196 men, 263 women, 5 unreported, mean age = 46.29, *SD* = 12.03). Most participants were full-time employees of companies, organizations, or agencies in Beijing, Shanghai, and Wuhan in China or Tokyo in Japan. Also, there were few business owners in those locations. They were opportunity samples, so the researchers did not target specific companies or job types.

### Materials and procedure

The questionnaire used in the study consisted of four sections: a face sheet, Beck Depression Inventory-II (BDI-II), a sleep disturbance questionnaire, and a job stressor assessment questionnaire. The face sheet asked for participants’ age, gender, job description (free answer), and the presence of pharmacological treatments for sleep disturbances (yes or no).

BDI-II [47] consists of 21 self-report measuring items, asking participants the severity of depression in the latest one-month period. Each item addresses one of the following symptom categories: depressive thoughts (e.g., pessimism, suicidal thoughts or wishes, worthlessness), feelings (e.g., sadness, guilty feelings, punishment feelings), behaviors (e.g., crying, indecisiveness), and physical conditions (e.g., changes in sleeping patterns and apatite, fatigue). Four choices representing varying degrees of symptom severity (e.g., 0. I do not feel sad; 1. I feel sad; 2. I am sad all the time and I can’t snap out of it; 3. I am so sad or unhappy that I can’t stand it) were used for responses. A score of 0 to 3 is allocated to each choice, making the total score range from 0 to 63. Existing Chinese [48] and Japanese [49] versions of this scale were used. In terms of this scale’s reliability, several studies reported high internal consistency, with Cronbach’s *α* above .80 [49–51]. The test-retest reliability correlation was also reported to be high (*r* = .07 in Kühner et al. [51]).

The sleep disturbance questionnaire in this study was identical to the one used by Matsuda and Kikutani [24]. It contained 13 items asking participants about the symptoms of insomnia, hypersomnia, and nightmares experienced in the last month. Eight items measuring insomnia were taken from the Athene Insomnia Scale (AIS) [52]. Established Chinese [53] and Japanese [54] versions were used. The first five items of AIS assess the quality of sleep as well as difficulties in getting asleep and maintaining sleep. The rest of the items assess the consequences of insomnia the next day. Each question states a topic (e.g., Awakenings during the night) accompanied by four answer choices (e.g., 0. No problem; 1. Minor problem; 2. Considerable problem; 3. Serious problem or did not sleep at all). The score for each answer varies from 0 to 3 (0 always indicates no problem); thus, maximum severity is indicated by the total score of 24 (3 times 8 questions). A total score exceeding 6 is regarded as clinical insomnia [55]. In the original English version of AIS, the internal consistency among the eight items was high (Cronbach’s *α* = 0.90), and so was the test-retest reliability correlation coefficient (*r* = 0.90) at a one-week interval [52].

Two items related to hypersomnia and three for nightmares were created based on the diagnostic criteria of ICD-10 [56]. The hypersomnia items asked participants whether they experienced prolonged main sleep episodes caused by difficulty waking and whether such sleep disturbance negatively impacted their daily lives. The nightmare items asked participants whether they experienced the recurrence of extremely distressful dreams, alertness after awakening from the nightmare, and significant distress or impairment in life caused by the nightmare. These questions were answered using 0 to 3 scales identical to the AIS; therefore, the maximum score for hypersomnia was 6, and that for nightmare was 9. The Chinese and Japanese versions of the hypersomnia and nightmare items were taken from the relevant language editions of the ICD-10.

The presence of stressors was measured using the Brief Job Stress Questionnaire (the BJSQ) created by the Japanese Ministry of Health, Labor, and Welfare [57]. This questionnaire is available in several different languages, and the English, Japanese, and Chinese versions are included in supplemental materials (S1 File). The original was in Japanese, and the Chinese version has been validated recently [58]. The questionnaire contains a total of 57 items, assessing the presence of stressors at the workplace (17 items), stress responses in the past month (28 items), available social support (9 items), and the level of satisfaction with the job and family life (2 items). Participants selected 1 (Very much so) to 4 (Not at all) for each statement to indicate whether it applied to them. Among the 57 BJSQ items, those measuring the presence of stressors and available social support were analyzed.

### Data collection and ethical considerations

The survey was transformed into an online questionnaire to be distributed. Participants were recruited either directly or indirectly by the authors. Directly recruited participants received an email explaining the research purpose and showing the study link from the authors. Some of them who were directly contacted were office managers of companies, and they passed the study link to their colleagues and staff. The majority of the responses were collected with the help of those managers. The survey was conducted anonymously from April to June 2017, and all participants were treated in accordance with the ethical guidelines of the American Psychological Association. The first page of the survey explained the research purpose and ethical considerations. It asked participants to indicate whether they give their consent to the study by choosing "agree to participate" or "disagree to participate". The survey ended there if no consent was given. The present study did not include any minors or children who would require parents’ or guardians’ consent for participation. In order to keep the study anonymous, participants were not asked to report the name of their employer. Due to the anonymity of the survey, the authors cannot identify individual participants from the data. The research was approved by the ethics committee of the Graduate School of Sociology at Toyo University (No. P16015, approved on December 12^th^, 2016).

### Data analysis

The current study mainly used structural equation modeling (SEM), and the target sample size was set as twenty times the number of measured variables, which is often used as a required size for SEM [59]. There were six measured variables (BDI-II, job stress, social support, insomnia symptoms, hypersomnia symptoms, and nightmare symptoms), and nationality can also be counted as a variable. Thus, 140 participants per country were aimed as a minimum sample size.

For the gathered data, the summed scores of the six variables were calculated first, and an independent sample t-test was performed for each variable to reveal differences between the Chinese and Japanese groups. Next, a theoretically hypothetical model of depression was tested using SEM. The hypothetical model contained "severity of depression (DBI-II)" as the dependent variable and "job stress," "social support," and "sleep disturbance" as predictor variables. Depression, job stress, and social support were observed variables, while sleep disturbance was a latent variable defined by the combination of the scores for insomnia, hypersomnia, and nightmares. The model testing involved SEM with the maximum likelihood estimates, which was analyzed by IBM SPSS Amos 27. The model supposes that life stressors increase sleep disturbances, consequently impacting depression. It means that the stressor would directly predict the extent of depression, but its indirect effect through sleep disturbance would also be significant. The indices for the model fitness were *X*^*2*^, the goodness of fit statistic (GFI), adjusted goodness of fit statistic (AGFI), comparative fit index (CFI), standardized root mean square residual (SRMR), and root mean square error of approximation (RMSEA). For these indices, the following cutoffs were employed to judge the goodness of the model: *χ*^*2*^/*df* ≤ 5, GFI ≥ .90, AGFI ≥ .90, CFI ≥ .95, SRMR ≤ .05, RMSEA ≤ .06 [60].

The theoretical basis of the model is the evidence showing that sleep problems, especially insomnia, significantly predict depression [7–20] and that stress level does not predict future depression onset effectively in some cases [23]. Stress and sleep disturbances must be strongly related, and considering the findings by Feldman et al. [23], it is reasonable to assume that life stress can increase depressive symptoms indirectly by worsening sleep disturbances. The current hypothetical model was identical to that in the research by Matsuda and Kikutani [24], which fitted their data well.

## Results

### Comparison between the Chinese and Japanese workers for depression, stressors, social support, and sleep disturbance symptoms

According to the free-answer job description question, participants had various jobs. Also, a significant number of participants did not answer this question. Therefore, the present research did not control the job in the analyses. Nevertheless, the job descriptions were categorized based on the International Standard Classification of Occupations [61]. The number of participants in each job category was reported in the supplemental material (S1 Table). Regarding the companies for which participants worked, no information was available for analysis since they did not reveal it. However, it can be speculated that majority of Chinese participants worked for national companies for utility, because many of the office managers who helped the data collection worked for those companies. Majority of Japanese participants were likely to be workers in large private companies. Japanese companies are categorized in three groups, large, middle, and small. Large companies should have more than 1000 employers.

The summed scores of BDI-II, the presence of job stress (the 17 items about the presence of stressors at the workplace in the BJSQ), available social support (the 9 items about social support in the BJSQ), and the symptoms of the three sleep disturbances for each participant were calculated and used as dependent variables. The response for each BJSQ item was re-coded using the four-point scale (1 to 4) to make the higher scores indicate higher stress levels. The items about social support were treated similarly to make the higher scores indicate more support. No gender difference was found for all those variables, so all the reported analyses were performed on the gender-combined data. Table 1 reports the mean and standard deviation (in brackets) of those variables as well as their Skewness and Kurtosis values. All those variables met the normal distribution criteria of Skewness ranging between -2 to +2 and kurtosis being between -7 to +7 [62, 63]. The inter-item reliability (Cronbach’s *α*) for the measured scales were as follows: BDI-II, Chinese (C) = .918, Japanese (J) = .904; the stressors, C = .696, J = .785; the social support, C = .862, J = .861; the sleep disturbance, C = .805, J = .736. An independent sample t-test was performed for each variable in Table 1 to reveal differences between the Chinese and Japanese groups.

**Table 1:** The means (*SD*) of the measurements for Chinese and Japanese participants, their distribution indices (*SE*), and the t-test results.

	Chinese (*n* = 185)	Japanese (*n* = 464)	Distribution (*n* = 649)		*t* (647)	*p*
	Mean (*SD*)	Mean (*SD*)	Skewness (*SE*)	Kurtosis (*SE*)		
Depression (BDI-II)	9.85 (9.64)	9.16 (7.54)	1.37 (.10)	2.11 (0.19)	-.97	.332
Stressors	40.68 (7.00)	39.15 (7.17)	-.07 (.10)	-.03 (.19)	-2.46	.014
Social support	24.37 (5.66)	26.44 (5.14)	-.06 (.10)	-.54 (.19)	4.49	< .001
Insomnia	4.97 (3.55)	4.25 (3.00)	1.11 (.10)	1.53 (.19)	-2.61	.009
Hypersomnia	1.17 (1.04)	0.80 (0.96)	1.16 (.10)	1.59 (.19)	-4.29	< .001
Nightmare	2.46 (1.84)	1.26 (1.35)	.66 (.10)	-.47 (.19)	-9.23	< .001

No group difference was found for the severity of depression. However, the Chinese participants reported significantly higher levels of stress than the Japanese. At the same time, the reported social support was less for the Chinese. There were significant group differences in the sleep disturbance scores, and the means were consistently higher for the Chinese than for the Japanese.

### Assessment of the depression model

**Fig 1:**
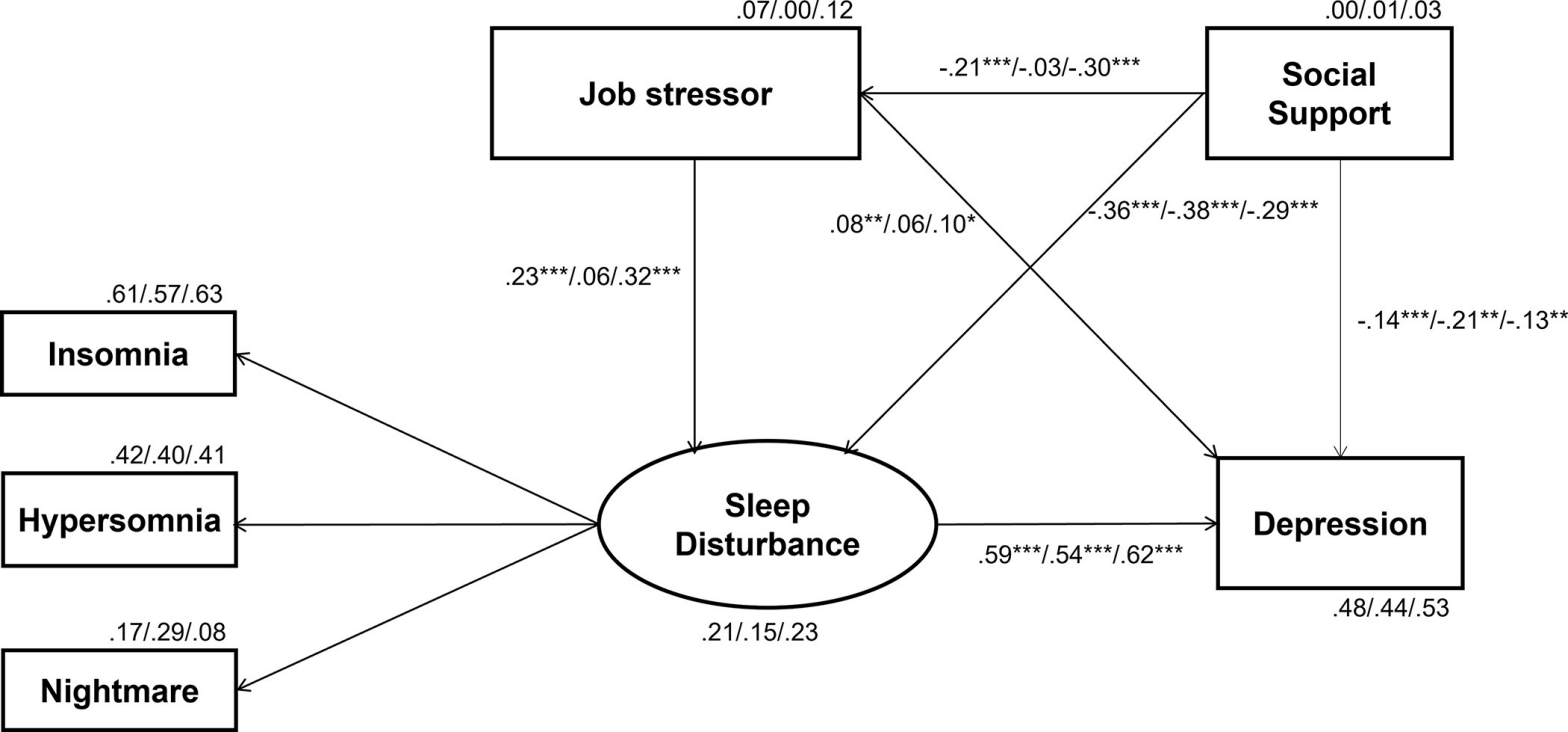
The depression model for all data, Chinese data, and Japanese data. The reported values are standardized coefficients in the order of All/Chinese/Japanese. The asterisks indicate as follows: *p < .05, **p < .01, ***p < .001.

The hypothetical model of depression was tested using SEM. The dependent variable was "severity of depression", and the predictor variables were "job stress," "social support," and "sleep disturbance". The regression weight for the path from sleep disturbance to insomnia was set to 1. This model focuses on whether the stressor would indirectly predict depression through sleep disturbance (see Fig 1). Since the mean age of Chinese and Japanese samples differed by more than 10 years, participants’ age was included in the model as a control variable. It means that paths were drawn from age to all the other observed variables, while these paths were omitted from Fig 1. Adding this control variable changes the minimum sample size from 140 to 160 per country, but the current data met this criterion. A further sample size calculation for this model was performed using an online sample size calculator for SEM (Daniel Soper sample size calculator, https://www.danielsoper.com/statcalc/calculator.aspx?id=89). The site revealed that the minimum sample size for this model, which has five variables (severity of depression, job stress, social support, sleep disturbance, and age) measured through a total of 61 items (21, 17, 9, 13, and 1 respectively), is 150 for observing medium size effects (Cohen’s *d* = 0.3) with power level of 0.8. Therefore, the current sample of 185 Chinese and 464 Japanese was considered sufficient.

The model was first assessed for all data without the distinction of nationality. The result was a good fit (*X*^*2*^ = 14.57, *df* = 6, *p* = .024, *χ*^*2*^/*df* = 2.43, GFI = .99, AGFI = .97, CFI = .99, SRMR = .02, RMSEA = .05). Next, it is examined whether this hypothetical model was superior to other theoretically sound nested models. The first alternative model (Alternative 1) assumes no relationship between job stress and sleep disturbance. The fitness indicators were as follows: *X*^*2*^ = 39.34, *df* = 7, *p* < .000, *χ*^*2*^/*df* = 5.62, GFI = .98, AGFI = .93, CFI = .96, SRMR = .05, RMSEA = .08. The next alternative model (Alternative 2) was almost identical to Alternative 1, but the direction of the path between the sleep disturbance and depression was reversed. This model represents the mechanism that job stress increases depressive symptoms, and sleep disturbance manifests as a symptom of depression. The fitness indicators of Alternative 2 were better than Alternative 1 (*X*^*2*^ = 20.72, *df* = 7, *p* < .004, *χ*^*2*^/*df* = 2.96, GFI = .99, AGFI = .96, CFI = .99, SRMR = .03, RMSEA = .06). Finally, the hypothetical model was compared with Alternative 2, using the likelihood ratio test [see 64]. The test compared the two models’ deviance information criteria (DIC) using *χ*^*2*^ distribution. The DIC and the effective number of parameters were determined through Bayesian estimates (sample size of 100,000). The hypothetical model’s DIC was 72.63, and the effective number of parameters was 29. The DIC of Alternative 2 was 77.05, and its effective number of parameters was 28. The DIC difference (4.42) with 1 degree of freedom (the difference in parameter numbers) exceeded the critical value of 3.84, indicating that the fitness level of these two models significantly differed. Based on the fitness indicators, the hypothetical model was judged to be the better model.

Fig 1 reports the hypothetical model structure for all data, Chinese data, and Japanese data in one figure. Three separate figures can be found in the supplemental material (S2 File). The crucial aspect of this model is that job stress influences depression symptoms indirectly through sleep disturbances. The result showed that the direct (*β* = .08, *p* = .035, 95% CI = .007 to .171) and indirect effects (*β* = .13, *p* = .002, 95% CI = .077 to .205) of job stress on depression were both statistically significant, but the effect was stronger for the latter. The model also shows that social support can mediate depressive symptoms, job stress, and sleep disturbances.

Next, the hypothetical model was assessed by nationality using a multi-group path analysis. The fitness indices met the criteria of a good fit model (*X*^*2*^ = 28.67, *df* = 18, *p* = .053, *χ*^*2*^/*df* = 1.59, GFI = .99, AGFI = .97, CFI = .99, SRMR = .02, RMSEA = .02). However, the analysis revealed slightly different pictures for the Japanese and Chinese data. While the Japanese result was identical to the overall one, the Chinese model showed few contrasts. For the Chinese participants, although sleep disturbances were strongly associated with the severity of depression, job stressor was related to none of the variables. The Chinese result indicates that social supports moderate the severity of depression and sleep disturbance symptoms, but they do not influence job stress.

The source of the difference between the Chinese and Japanese results might be the response patterns for the job stress questionnaire, which consisted of 17 items. In order to compare the response patterns between the two groups of participants, the mean score of each job stress question was calculated for the Chinese and Japanese groups. Then, a correlation coefficient of the 17 mean scores was examined. The scores between the two participant groups were highly correlated (*r* = .771, *p* < .001). Therefore, it is unlikely that Chinese participants answered those questions very differently from the Japanese.

## Discussion

The present research targeted employees in China and Japan to examine the relationship between depression, job stress, and sleep disturbance. It was hypothesized that job stress would impact depressive symptoms through increasing sleep disturbance, and this indirect effect is stronger than its direct effect on depression. This hypothesis was supported by the study with university students conducted by Matsuda and Kikutani [24], and similar results were found in the present study with the working population.

The results suggest that treating sleep disturbance is an effective solution to mediate depressive symptoms, and the impact of the treatment can be as great as treatment for stress reduction. Working on sleep disturbance might be easier than alleviating stressors since the work environment is hard to control for individual employees. In contrast, people have more control over their sleeping habits and environment. Thus, it is essential to inform the public that treating sleep disturbance is urgent once they start having depressive symptoms. An increasing amount of evidence shows that sleep disturbance can cause depression rather than being one of its symptoms [7, 14, 15, 19]. However, a common notion is that stress triggers depression, and sleep disturbances are just symptoms. Thus, most people tend to prioritize dealing with stress. Of course, reducing stress is an important aspect of depression treatment, but the present research strongly suggests that treating sleep disturbance should be very beneficial.

The importance of addressing sleep disturbance goes beyond depression treatment. As Chattu et al. [65] reviewed, insufficient sleep is associated with a wide range of adverse health and social outcomes, and sleep disturbance is a serious international health problem. It is estimated that sleep disturbance affects at least 20% of the general population [66], and adults in Japan [67] and China [68] are no exception. Sleep problems among Japanese workers are also reported frequently [36, 37, 69, 70]. Interestingly, the Chinese participants in the present research reported higher sleep disturbance scores. It may reflect the overwork among Chinese people, represented by the 996 working regime, which requires employees to work 9 am to 9 pm, 6 days a week. This regime is widespread in China and is known to prevent many workers from having healthy lifestyles [71]. Whether the participants were working under this regime is unknown in the present research, but it is a possible cause for the Chinese participants to have worse sleeping habits than the Japanese. It may also be related to the Chinese participants’ higher job stress in the present study.

Interestingly, however, the results showed that the relationship between job stress and sleep disturbance was different between the two groups of participants. For the Chinese, job stress was not associated with depression or sleep disturbance symptoms nor moderated by available social support. In contrast, job stress was related to depression, sleep disturbance, and social support for the Japanese. This discrepancy might have resulted from the relatively small sample size of the Chinese (185 Chinese, contrasting to 464 Japanese). However, the path between job stress and depression did not become significant even when the analysis employed a boot-strapping method to increase the sample size to 500. The Chinese version of the job stress questionnaire has been validated [58], and the correlation between the Chinese and Japanese data for mean stress scores was high. The mean values and standard deviations of the two groups were also very similar, although the Chinese means were slightly higher. Therefore, it is unlikely that Chinese participants misunderstood questions or responded wrongly. This discrepancy of the result was discussed further in the next section in relation to research limitations.

The present findings demonstrated the positive impact of social support, which reduces stress and improves well-being. Chinese participants reported a lesser extent of available social support than Japanese participants. However, the current result does not necessarily mean that social support is more difficult to obtain in China than in Japan because the perception of receiving social support is strongly influenced by the subjective evaluation of how much social support the person gives to others [72]. Nevertheless, the hypothetical model of the present research suggests that having social support helps reduce depressive symptoms, job stress, and sleep disturbance. Therefore, it is crucial for employers to create a working environment in which workers can support each other. Similarly, employers must ensure that their workers have enough sleep [65]. Avoiding to give them long working hours must be effective [33]. Also, business owners should strive to diminish their employees’ stress by discouraging conflict in the workplace [37] and reducing psychological demands for work [34].

### Implications of cultural differences and research limitations

The present research revealed statistically significant differences between Chinese and Japanese data for job stress, social support, and sleep disturbance. Despite those differences, culturally invariant depression mechanisms were apparent, such as the strong relationship between depressive symptoms and sleep disturbances as well as the positive influence of social support on depression and sleep disturbances. It is meaningful to reveal such core element of psychological illness through cross-cultural research. On the contrary, attributing the group differences in dependent measures to culture is difficult, and interpretation of the results requires great deal of cautions due to many potential confounding variables. For example, how people conceptualize mental illnesses seems to be influenced by culture. In case of depression sypmtoms, some researchers argue that Chinese people tend to emphasize physical impairment and pain more than other syptoms related to their psychology, such as mood and motivation [73]. Response bias tendenceis for questionnaires (e.g., preferring extreme answers or middle ones) are also found to be different across cultures [74]. Without controlling those confounding variables, it is difficult to draw strong conclusions on cultural influences on depression, and unfortunately, the present research contains several limitations in terms of variable control and sample characteristics.

Regarding the variable control, the study did not collect detailed information about participants’ working styles, such as hours, location, tasks, and environment. Working styles vary immensely across jobs, and specific jobs may be prone to specific risk factors for depression. However, this research cannot explore such issues due to the insufficient control of work-related variables. For example, working hours can significantly impact sleeping habits. It is reported that shift workers tend to suffer from insufficient sleep [75], and some participants in the current study worked as nurses and doctors who might have had working shifts. Although identifying job-specific mechanisms of depression was not the main aim of this research, the detailed information related to work should have been collected more carefully and controlled in the analysis. Detailed information about participants’ employer was also missing in the present study. Nature of the companies they worked for, such as size, business sectors, and business styles (e.g., public/private, national/international), likely to influence cooperate culture and working environments. So, such information should be gathered in future studies.

In addition to the job-related details, family-related information, such as family structure and living arrangements, should be more carefully controlled in the future because these issues must greatly impact social support and sleeping habits. Family members living with participants can be a source of social support but some members, such as small children, can disturb good sleeping habits. The lack of variable control in the present study may underlie some of the results that are hard to interpret, such as the lack of relationship between job stress and depression in the Chinese sample. If their sleep disturbance is predominantly caused by child rearing, for example, their job stress will be only weakly related with sleep disturbance and depression. Measuring possible confounding variables is essential to interpret results using cultural characteristics.

The generalizability of the present finding is also limited. The study’s sample size was fairly small, considering the vast population of the two countries. The unbalanced sample sizes between the two cultural groups were especially undesirable. The reason for the relatively small Chinese sample size is the authors’ limited accessibility to Chinese workers, and it might have led to contrasting results found for the Chinese and Japanese data. Importantly, the Chinese and Japanese samples have met the requirement for the minimum sample size to run SEM analysis, so the model should apply to the general population to a certain extent, especially to people with similar characteristics to the present sample, namely, company employees in large cities. The finding about the strong connection between sleep disturbance and depressive symptoms was consistent across the two countries, and similar evidence has been reported in the past [7, 8, 23, 24]. Thus, this part of the result should be generalizable to other cultures’ populations. However, the study did not include jobs in rural areas such as farming, and a large number of people in the two countries live outside cities. Therefore, further investigations with a wider variety of jobs must be conducted to verify the present findings. Also, people with the same occupation can have very different work environments depending on the culture. Thus, future cross-cultural research should be carefully designed to focus on particular occupations or include various jobs to see general tendencies. In addition, it is desirable to increase the number of cultures investigated at the same time. Comparing just two countries is often insufficient to determine whether a difference in a behavior is caused by a difference in some cultural characteristic because the correlation between the behavior and cultural characteristic can be spurious. Therefore, it is recommended to include three or more cultures with varying levels of the behavior and cultural characteristic and see whether the correlation is always observed [see 76]. New studies without the limitations of the present study will enhance our understanding of the relationship between depression and sleep disturbance.

## Supporting information

S1 File The brief job stress questionnaire in English, Chinese and Japanese. The 57-item questionnaire created by the Japanese Ministry of Health, Labor, and Welfare.
(DOC)

S2 File The results of the model analysis described by nationality. (DOCX)

S1 Table Participants’ jobs classified in accordance with ISCO-08. (DOCX)
