# Supplementary material for: Impacts of sleep disturbance and work-related life stress on depression among Japanese and Chinese workers
Source: PLoS One. 2024 Jun 27;19(6):e0305936. doi: 10.1371/journal.pone.0305936 (PMC11210821; doi:10.1371/journal.pone.0305936)
Supplement: S1 File — The 57-item questionnaire created by the Japanese Ministry of Health, Labor, and Welfare. (DOC) [file pone.0305936.s001.doc]

**The Brief Job Stress Questionnaire English version**

**Please answer the following questions concerning your job by circling the number that best fits your situation.**

| Very much so | Moderately so | Somewhat | Not at all |
| --- | --- | --- | --- |

1. I have an extremely large amount of work to do 1 2 3 4
2. I can't complete work in the required time 1 2 3 4
3. I have to work as hard as I can 1 2 3 4
4. I have to pay very careful attention 1 2 3 4
5. My job is difficult in that it requires a high level of knowledge and technical skill 1 2 3 4
6. I need to be constantly thinking about work throughout the working day 1 2 3 4
7. My job requires a lot of physical work 1 2 3 4
8. I can work at my own pace 1 2 3 4
9. I can choose how and in what order to do my work 1 2 3 4
10. I can reflect my opinions on workplace policy 1 2 3 4
11. My knowledge and skills are rarely used at work 1 2 3 4
12. There are differences of opinion within my department 1 2 3 4
13. My department does not get along well with other departments 1 2 3 4
14. The atmosphere in my workplace is friendly 1 2 3 4
15. My working environment is poor (e.g. noise, lighting, temperature, ventilation) 1 2 3 4
16. This job suits me well 1 2 3 4
17. My job is worth doing 1 2 3 4

**Please answer the following questions concerning your health during the past month by circling the number that best fits your situation.**

| Almost never | Sometimes | Often | Almost always |
| --- | --- | --- | --- |

18. I have been very active 1 2 3 4

19. I have been full of energy 1 2 3 4

20. I have been lively 1 2 3 4

21. I have felt angry 1 2 3 4

22. I have been inwardly annoyed or aggravated 1 2 3 4

23. I have felt irritable 1 2 3 4

24. I have felt extremely tired 1 2 3 4

25. I have felt exhausted 1 2 3 4

26. I have felt weary or listless 1 2 3 4

27. I have felt tense 1 2 3 4

28. I have felt worried or insecure 1 2 3 4

29. I have felt restless 1 2 3 4

30. I have been depressed 1 2 3 4

31. I have thought that doing anything was a hassle 1 2 3 4

32. I have been unable to concentrate 1 2 3 4

33. I have felt gloomy 1 2 3 4

34. I have been unable to handle work 1 2 3 4

35. I have felt sad 1 2 3 4

36. I have felt dizzy 1 2 3 4

37. I have experienced joint pains 1 2 3 4

38. I have experienced headaches 1 2 3 4

39. I have had a stiff neck and / or shoulders 1 2 3 4

40. I have had lower back pain 1 2 3 4

41. I have had eyestrain 1 2 3 4

42. I have experienced heart palpitations or shortness of breath 1 2 3 4

43. I have experienced stomach and / or intestine problems 1 2 3 4

44. I have lost my appetite 1 2 3 4

45. I have experienced diarrhea and / or constipation 1 2 3 4

46. I haven’t been able to sleep well 1 2 3 4

**Please answer the following questions concerning satisfaction by circling the number that best fits your situation.**

| Extremely | Very much | Somewhat | Not at all |
| --- | --- | --- | --- |

How freely can you talk with the following people?

1. Superiors 1 2 3 4
2. Co-workers 1 2 3 4
3. Spouse, family, friends, etc. 1 2 3 4

How reliable are the following people when you are troubled?

1. Superiors 1 2 3 4
2. Co-workers 1 2 3 4
3. Spouse, family, friends, etc. 1 2 3 4

How well will the following people listen to you when you ask for advice on personal matters?

1. Superiors 1 2 3 4
2. Co-workers 1 2 3 4
3. Spouse, family, friends, etc. 1 2 3 4

**Please answer the following questions concerning satisfaction by circling the number that best fits your situation.**

| Satisfied | Somewhat satisfied | Somewhat dissatisfied | Dissatisfied |
| --- | --- | --- | --- |

1. I am satisfied with my job 1 2 3 4
2. I am satisfied with my family life 1 2 3 4
3. **The Brief Job Stress Questionnaire Chinese version**

职业性压力简易调查问卷（57项）

1 是 2大概是 3不全是 4不是

A 在下列问题中，请在最符合您工作情况的选项上画圈。

1. 必须做大量的工作 1 2 3 4
2. 不能按时完成工作 1 2 3 4
3. 不得不拼命工作 1 2 3 4
4. 工作时需要高度集中注意力 1 2 3 4
5. 工作艰巨并且需要高度的知识和技术 1 2 3 4
6. 上班时工作的事情片刻都不能离开脑海 1 2 3 4
7. 需要消耗大量体力的工作 1 2 3 4
8. 自己可以掌握工作的进度 1 2 3 4
9. 自己可以决定工作内容的顺序和工作方法 1 2 3 4
10. 自己的意见在单位的工作方针中能够得到反映 1 2 3 4
11. 自己具备的技能和知识很少能在工作中发挥 1 2 3 4
12. 自己所在的工作部门内存在意见分歧 1 2 3 4
13. 自己所在的工作部门和其他的工作部门有不合的地方 1 2 3 4
14. 单位的工作氛围友好 1 2 3 4
15. 单位的工作环境（噪音、照明、温度、换气等）不好 1 2 3 4
16. 工作内容适合自己 1 2 3 4

17.从事的工作有意义 1 2 3 4

B 请问您最近一个月的下列状况，请在最符合的状况的选项上画圈。

1 几乎没有 2 偶尔有 3经常有 4 几乎总有

1. 感到精神饱满 1 2 3 4
2. 浑身充满活力 1 2 3 4
3. 生气勃勃 1 2 3 4
4. 感到愤怒 1 2 3 4
5. 心里生闷气 1 2 3 4
6. 情绪烦躁 1 2 3 4
7. 十分疲劳 1 2 3 4
8. 精疲力尽 1 2 3 4
9. 疲惫乏力 1 2 3 4
10. 精神一直处于紧张状态 1 2 3 4
11. 感到不安 1 2 3 4
12. 静不下心来 1 2 3 4
13. 感到忧郁 1 2 3 4
14. 做什么都嫌麻烦 1 2 3 4
15. 对事对物无法集中精力 1 2 3 4
16. 心情不愉快 1 2 3 4
17. 无法着手工作 1 2 3 4
18. 感到悲伤 1 2 3 4
19. 感到头晕眼花 1 2 3 4
20. 全身各处关节疼痛 1 2 3 4
21. 感到头重头痛 1 2 3 4
22. 颈部或肩部有僵硬感 1 2 3 4
23. 感到腰痛 1 2 3 4
24. 感到眼睛疲劳 1 2 3 4
25. 有心悸或气短 1 2 3 4
26. 胃肠不适 1 2 3 4
27. 没有食欲 1 2 3 4
28. 有便秘或腹泻 1 2 3 4
29. 睡眠不好 1 2 3 4

C 在您周围的人员中，请在您认为最适合的选项上画圈。

1非常可以 2相当可以 3多少可以 4完全不可以

您可以与下列人员进行随意交谈吗？

1. 上司 1 2 3 4
2. 同事 1 2 3 4
3. 配偶、家人、朋友等 1 2 3 4

您遇到困难时、下列人员是否可以信赖？

1. 上司 1 2 3 4
2. 同事 1 2 3 4
3. 配偶、家人、朋友等 1 2 3 4

因个人的问题需要咨询时，下列人员是否可以与之商量？

7. 上司 1 2 3 4

8.同事 1 2 3 4

1. 配偶、家人、朋友等 1 2 3 4

D 有关满足度

1满足 2还算满足 3稍有不满 4不满

1. 对工作感到满足 1 2 3 4
2. 对家庭生活感到满足 1 2 3 4

**The Brief Job Stress Questionnaire Japanese version**

職業性ストレス簡易調査票（57項目）

**Ａ　あなたの仕事についてうかがいます。最もあてはまるものに○を付けてください。**

1．そうだ　2．まあそうだ　3．ややちがう　4．ちがう

　1. 非常にたくさんの仕事をしなければならない-------------------- １　　２　　３　　４

　2. 時間内に仕事が処理しきれない-------------------------------- １　　２　　３　　４

　3. 一生懸命働かなければならない-------------------------------- １　　２　　３　　４

　4. かなり注意を集中する必要がある------------------------------ １　　２　　３　　４

　5. 高度の知識や技術が必要なむずかしい仕事だ-------------------- １　　２　　３　　４

　6. 勤務時間中はいつも仕事のことを考えていなければならない------ １　　２　　３　　４

　7. からだを大変よく使う仕事だ---------------------------------- １　　２　　３　　４

　8. 自分のペースで仕事ができる---------------------------------- １　　２　　３　　４

　9. 自分で仕事の順番・やり方を決めることができる---------------- １　　２　　３　　４

　10. 職場の仕事の方針に自分の意見を反映できる-------------------- １　　２　　３　　４

　11. 自分の技能や知識を仕事で使うことが少ない-------------------- １　　２　　３　　４

　12. 私の部署内で意見のくい違いがある---------------------------- １　　２　　３　　４

　13. 私の部署と他の部署とはうまが合わない------------------------ １　　２　　３　　４

　14. 私の職場の雰囲気は友好的である------------------------------ １　　２　　３　　４

　15. 私の職場の作業環境（騒音、照明、温度、換気など）はよくない-- １　　２　　３　　４

　16. 仕事の内容は自分にあっている-------------------------------- １　　２　　３　　４

　17. 働きがいのある仕事だ---------------------------------------- １　　２　　３　　４

**Ｂ　最近1 か月間のあなたの状態についてうかがいます。最もあてはまるものに○を付けてください。**

1．ほとんどなかった　2．ときどきあった　3．しばしばあった　4．ほとんどいつもあった

　1. 活気がわいてくる-------------------------------------------- １　　２　　３　　４

　2. 元気がいっぱいだ-------------------------------------------- １　　２　　３　　４

　3. 生き生きする------------------------------------------------ １　　２　　３　　４

　4. 怒りを感じる------------------------------------------------ １　　２　　３　　４

　5. 内心腹立たしい---------------------------------------------- １　　２　　３　　４

　6. イライラしている-------------------------------------------- １　　２　　３　　４

　7. ひどく疲れた------------------------------------------------ １　　２　　３　　４

　8. へとへとだ-------------------------------------------------- １　　２　　３　　４

　9. だるい------------------------------------------------------ １　　２　　３　　４

　10. 気がはりつめている------------------------------------------ １　　２　　３　　４

　11. 不安だ------------------------------------------------------ １　　２　　３　　４

　12. 落着かない-------------------------------------------------- １　　２　　３　　４

　13. ゆううつだ-------------------------------------------------- １　　２　　３　　４

　14. 何をするのも面倒だ------------------------------------------ １　　２　　３　　４

　15. 物事に集中できない------------------------------------------ １　　２　　３　　４

　16. 気分が晴れない---------------------------------------------- １　　２　　３　　４

　17. 仕事が手につかない------------------------------------------ １　　２　　３　　４

　18. 悲しいと感じる---------------------------------------------- １　　２　　３　　４

　19. めまいがする------------------------------------------------ １　　２　　３　　４

　20. 体のふしぶしが痛む------------------------------------------ １　　２　　３　　４

　21. 頭が重かったり頭痛がする------------------------------------ １　　２　　３　　４

　22. 首筋や肩がこる---------------------------------------------- １　　２　　３　　４

　23. 腰が痛い---------------------------------------------------- １　　２　　３　　４

　24. 目が疲れる-------------------------------------------------- １　　２　　３　　４

　25. 動悸や息切れがする------------------------------------------ １　　２　　３　　４

　26. 胃腸の具合が悪い-------------------------------------------- １　　２　　３　　４

　27. 食欲がない-------------------------------------------------- １　　２　　３　　４

　28. 便秘や下痢をする-------------------------------------------- １　　２　　３　　４

　29. よく眠れない------------------------------------------------ １　　２　　３　　４

**Ｃ　あなたの周りの方々についてうかがいます。最もあてはまるものに○を付けてください。**

1．非常に　2．かなり　3．多少　4．全くない

次の人たちはどのくらい気軽に話ができますか？

　1. 上司-------------------------------------------------------- １　　２　　３　　４

　2. 職場の同僚-------------------------------------------------- １　　２　　３　　４

　3. 配偶者、家族、友人等---------------------------------------- １　　２　　３　　４

あなたが困った時、次の人たちはどのくらい頼りになりますか？

　4. 上司-------------------------------------------------------- １　　２　　３　　４

　5. 職場の同僚-------------------------------------------------- １　　２　　３　　４

　6. 配偶者、家族、友人等---------------------------------------- １　　２　　３　　４

あなたの個人的な問題を相談したら、次の人たちはどのくらいきいてくれますか？

　7. 上司-------------------------------------------------------- １　　２　　３　　４

　8. 職場の同僚-------------------------------------------------- １　　２　　３　　４

　9. 配偶者、家族、友人等---------------------------------------- １　　２　　３　　４

**Ｄ　満足度について**

1．満足　2．まあ満足　3．やや不満足　4．不満足

　1. 仕事に満足だ------------------------------------------------ １　　２　　３　　４

　2. 家庭生活に満足だ-------------------------------------------- １　　２　　３　　４
