# Supplementary material for: Impacts of sleep disturbance and work-related life stress on depression among Japanese and Chinese workers
Source: PLoS One. 2024 Jun 27;19(6):e0305936. doi: 10.1371/journal.pone.0305936 (PMC11210821; doi:10.1371/journal.pone.0305936)

**S2 file: The results of the model analysis described by nationality.**


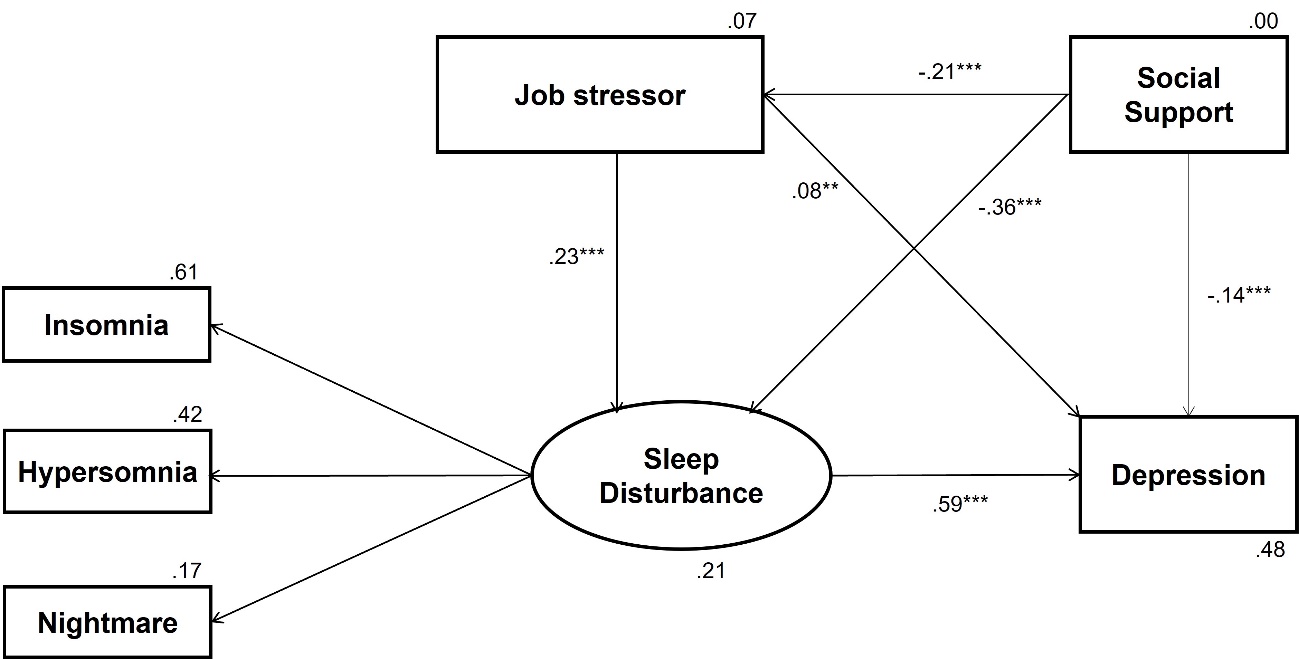


**Fig S1. The depression model for all data.** The asterisks indicate as follows: **p* < .05, ***p* < .01, ****p* < .001.


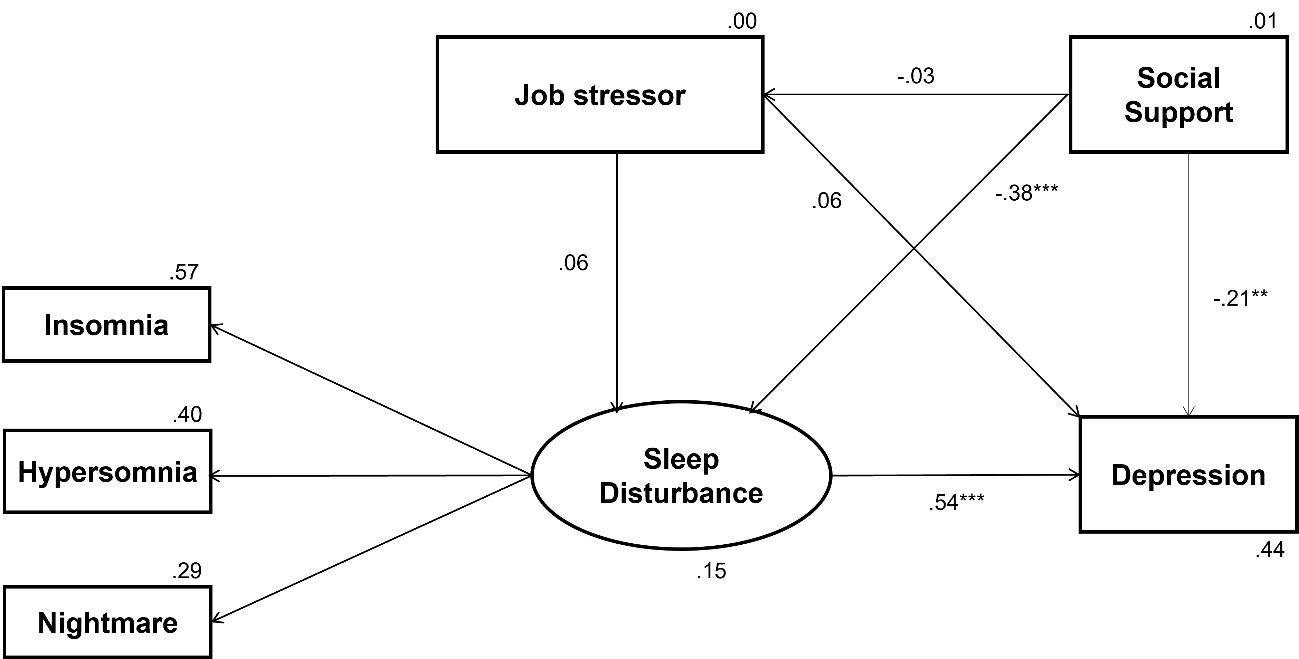


**Fig S2. The depression model for the Chinese data.** The asterisks indicate as follows: **p* < .05, ***p* < .01, ****p* < .001.

**Fig S3. The depression model for the Japanese data.** The asterisks indicate as follows: **p* < .05, ***p* < .01, ****p* < .001.


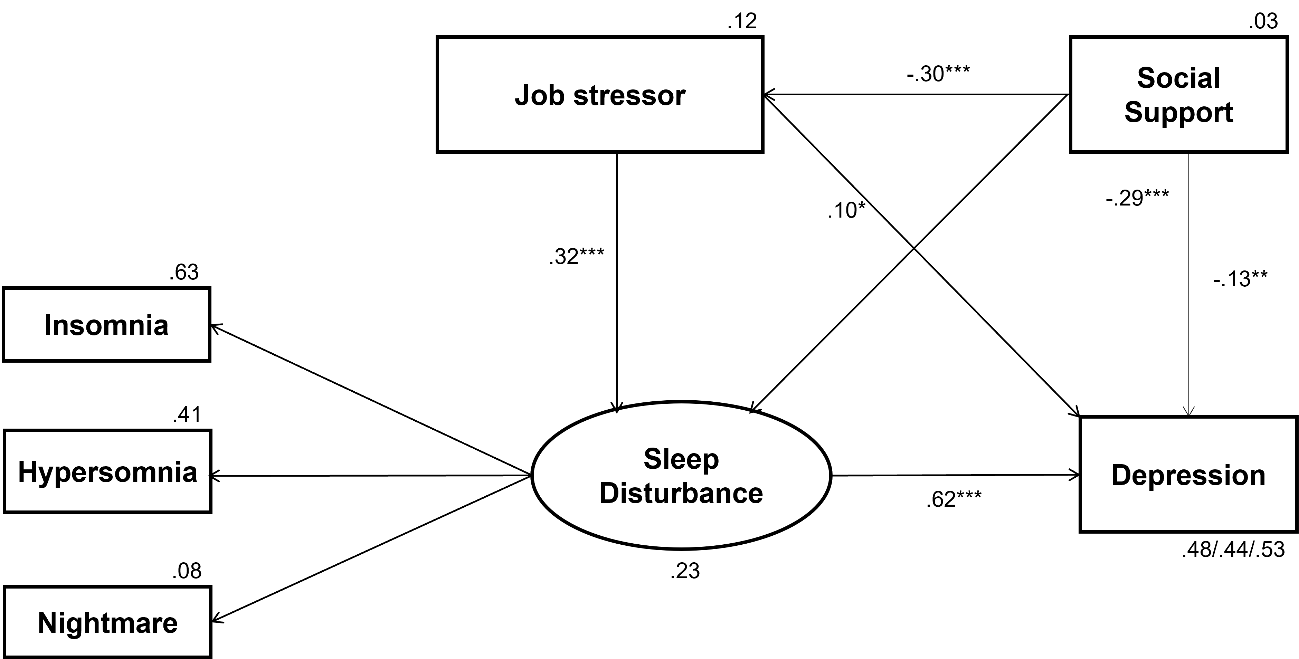

Supplement: S2 File — (DOCX) [file pone.0305936.s002.docx]
