# Supplementary material for: Impacts of sleep disturbance and work-related life stress on depression among Japanese and Chinese workers
Source: PLoS One. 2024 Jun 27;19(6):e0305936. doi: 10.1371/journal.pone.0305936 (PMC11210821; doi:10.1371/journal.pone.0305936)
Supplement: S1 Table — (DOCX) [file pone.0305936.s003.docx]

S1 Table. Participants’ jobs classified in accordance with ISCO-08

| Job category value on the SPSS data file |  | Chinese (*n*=185) | | Japanese (*n*=464) | |
| --- | --- | --- | --- | --- | --- |
|  |  | Number | Proportion | Number | Proportion |
| 1 | No response | 25 | 14% | 68 | 15% |
| 2 | Administration professionals | 18 | 10% | 19 | 4% |
| 3 | Architects, planners, surveyors and designers | 2 | 1% | 1 | 0% |
| 4 | Artistic, cultural and culinary associate professionals | 1 | 1% | 3 | 1% |
| 5 | Authors, journalists and linguists |  |  | 1 | 0% |
| 6 | Business and administration professionals |  |  | 3 | 1% |
| 7 | Business owners* |  |  | 9 | 2% |
| 8 | Business service agents |  |  | 1 | 0% |
| 9 | Child care workers and teachers' aides |  |  | 3 | 1% |
| 10 | Clerical support workers |  |  | 57 | 12% |
| 11 | Cooks |  |  | 2 | 0% |
| 12 | Electrotechnology engineers | 5 | 3% |  |  |
| 13 | Engineering professionals | 4 | 2% |  |  |
| 14 | Fashion industry* |  |  | 3 | 1% |
| 15 | Finance professionals | 9 | 5% |  |  |
| 16 | Food processing, wood working, game and other craft and related trades workers |  |  | 4 | 2% |
| 17 | General and keyboard clerks |  |  | 1 | 0% |
| 18 | Health professionals |  |  | 36 | 8% |
| 19 | Information and communications technology professionals |  |  | 51 | 11% |
| 20 | Laborers in mining, construction, manufacturing and transport | 14 | 8% | 3 | 1% |
| 21 | Legal professionals |  |  | 1 | 0% |
| 22 | Legal, social and religious associate professionals | 1 | 1% |  |  |
| 23 | Managers | 4 | 2% | 34 | 7% |
| 24 | Mathematicians, actuaries, and statisticians | 2 | 1% |  |  |
| 25 | Medical doctors |  |  | 5 | 1% |
| 26 | Nursing, and midwifery professionals |  |  | 13 | 3% |
| 27 | Office workers* | 39 | 21% | 25 | 5% |
| 28 | Part-time staffs* |  |  | 20 | 4% |
| 29 | Personal care workers in health services |  |  | 1 | 0% |
| 30 | Personal service workers |  |  | 7 | 2% |
| 31 | Plant and machine operators and assembles |  |  | 1 | 0% |
| 32 | Production department* | 15 | 8% |  |  |
| 33 | Professional |  |  | 2 | 0% |
| 34 | Sales workers |  |  | 1 | 0% |
| 35 | Sales, marketing and public relations professionals | 39 | 21% | 11 | 2% |
| 36 | Science and engineering professionals | 1 | 1% | 1 | 0% |
| 37 | Service and sales workers | 6 | 3% |  |  |
| 38 | Teaching professionals |  |  | 75 | 16% |
| 39 | Non-classifiable |  |  | 2 | 0% |

*Note.* The category with an asterisk is not included in ISCO-08
